# Fabrication of Infrared-Compatible Nanofluidic Devices for Plasmon-Enhanced Infrared Absorption Spectroscopy

**DOI:** 10.3390/mi11121062

**Published:** 2020-11-30

**Authors:** Thu Hac Huong Le, Takumi Matsushita, Ryoichi Ohta, Yuta Shimoda, Hiroaki Matsui, Takehiko Kitamori

**Affiliations:** 1 Department of Applied Chemistry, Graduate School of Engineering, The University of Tokyo, Tokyo 113-8654, Japan; matsu-tk@iis.u-tokyo.ac.jp; 2 Collaborative Research Organization for Micro and Nano Multifunctional Devices (NMfD), The University of Tokyo, Tokyo 113-8654, Japan; ohta@icl.t.u-tokyo.ac.jp (R.O.); kitamori@icl.t.u-tokyo.ac.jp (T.K.); 3 Department of Bioengineering/Electrical Engineering and Information systems, Graduate School of Engineering, The University of Tokyo, Tokyo 113-8654, Japan; shimoda@bioxide.t.u-tokyo.ac.jp (Y.S.); hiroaki@ee.t.u-tokyo.ac.jp (H.M.); 4 Department of Bioengineering, Graduate School of Engineering, The University of Tokyo, Tokyo 113-8654, Japan; 5 Institute of Nanoengineering and Microsystems iNEMS, Department of Power Mechanical Engineering, National Tsing Hua University, Hsinchu 300044, Taiwan

**Keywords:** nanofluidics, IR absorption spectroscopy, SEIRA, metamaterials, MIM perfect absorber

## Abstract

Nanofluidic devices have offered us fascinating analytical platforms for chemical and bioanalysis by exploiting unique properties of liquids and molecules confined in nanospaces. The increasing interests in nanofluidic analytical devices have triggered the development of new robust and sensitive detection techniques, especially label-free ones. IR absorption spectroscopy is one of the most powerful biochemical analysis methods for identification and quantitative measurement of chemical species in the label-free and non-invasive fashion. However, the low sensitivity and the difficulties in fabrication of IR-compatible nanofluidic devices are major obstacles that restrict the applications of IR spectroscopy in nanofluidics. Here, we realized the bonding of CaF_2_ and SiO_2_ at room temperature and demonstrated an IR-compatible nanofluidic device that allowed the IR spectroscopy in a wide range of mid-IR regime. We also performed the integration of metal-insulator-metal perfect absorber metamaterials into nanofluidic devices for plasmon-enhanced infrared absorption spectroscopy with ultrahigh sensitivity. This study also shows a proof-of-concept of the multi-band absorber by combining different types of nanostructures. The results indicate the potential of implementing metamaterials in tracking several characteristic molecular vibrational modes simultaneously, making it possible to identify molecular species in mixture or complex biological entities.

## 1. Introduction

As microfluidics and integrated micro chemical systems on-a-chip continue to show great performance and new functionalities in chemical and bioanalysis, synthesis, biosciences and technologies, there is an increasing demand for new robust and sensitive detection techniques. So far detections in microfluidics have so far mostly relied on optical techniques such as fluorescence or UV/visible absorption. However, UV and visible absorption lack chemical specificity, while fluorescence-based techniques suffer from labeling and photobleaching issues [1,2,3]. Vibrational spectroscopies, including infrared (IR) absorption and Raman spectroscopy, are one of the most powerful analytical tools, as they extract essential information of chemical bonds and molecular structures in the label-free and non-invasive fashion. They are especially useful in tracing subtle changes in the conformational structures of biomolecules in response to the surrounding environment or to in situ probing the kinetics of a chemical event. Molecular detection by Raman or IR spectroscopy in microfluidics, however, is quite challenging since they are extremely weak optical processes. Even though IR absorption is intrinsically more sensitive than Raman scattering by several orders of magnitude, the IR absorption cross-section is still very low in comparison with UV/visible absorption or fluorescence emission [4,5,6,7]. Recent advances in bright mid-IR light sources such as synchrotron radiation or tunable quantum cascade lasers (QCL), as well as mid-IR photon detectors have stimulated the development of highly sensitive Fourier-transformed infrared (FT-IR) spectromicroscopy towards microfluidic applications [8,9,10]. Another approach to overcome the low sensitivity in IR absorption spectroscopy is exploiting the plasmon resonance on a thin film, nanoparticle, or nanostructure of noble metals. This so-called surface enhanced infrared absorption (SEIRA) phenomenon involves the coupling of photon and vibrational modes of molecules when they are located in the enhanced electromagnetic field (i.e., hot-spots) of a propagating or localized surface plasmon polariton. It eventually leverages the interactions of molecules and photons, and consequently induces a significant enhancement in the IR absorption signal of molecules by 4–5 orders of magnitude [11,12]. Recently, the emergence of metamaterials—the field of artificial materials composed of metallo-dielectric periodic nanostructures—has offered a new degree of freedom to engineer hot-spots to further improve the performance of SEIRA [13,14].

Nanofluidics, the study and applications of fluids typically confined in structures of 1–1000 nm scale, have emerged recently in the footsteps of microfluidics with numerous novel fluidic functionalities and applications. In nanofluidics, certain effects are expected to become dominant due to the size reduction such as laminar flow, diffusion, surface area to volume ratio, or surface tension. Moreover, it has been confirmed that liquids confined on nanospaces exhibit specific properties which are not observed on microscale or in bulk. For example, nanoconfined water has unusual structural and dynamical properties such as higher viscosity, lower dielectric constant and refractive index, or higher proton mobility, compared to those in bulk [2,15]. Nanofluidics offers attractive platforms for chemical and bioanalyses by exploiting those effects. The implementation of nanofluidic devices in the analyses, however, is restricted by the lack of sensitive detection methods, since the number of molecules within a nanofluidic channel is very limited [15]. IR absorption spectroscopy in nanofluidic devices particularly is extremely difficult. Recently, the integration of metamaterial structures into nanofluidic channels has been demonstrated in the attempt to realize IR spectroscopy for micro/nanofluidics. This integration can take an explicit benefit of the enhanced light–matter interactions and the mass transport in the nanoscale fluidic channels, since the channel size is comparable to the characteristic lengthscale of the near-field plasmonic interactions [15,16]. For example, in previous studies, we have introduced a device configuration consisting of a nanofluidic channel with a depth of several tens of nanometers sandwiched between an array of nanostructures (i.e., plasmonic resonators) and a thick metal film (i.e., mirror film) [17,18,19]. Herein, the nanochannel performs as an insulator thin film, and together with the nanostructures and metal film, they form the well-known metal-insulator-metal (MIM) perfect absorber metamaterial structure. The device exhibits a quadrupole plasmon resonant mode, at which the enhanced field is localized in the nanofluidic gap defined by the nanostructures and the mirror film. Notably, this device allows the introduction of target molecules into the most enhanced electromagnetic field of MIM structures, resulting in a 105-fold improvement in the sensitivity compared to state-of-the-art SEIRA methods employing conventional MIM ones. We also succeeded in measuring the absorption of water confined in a ~10 nm gap, which corresponds to merely ~30 layers of water molecules [18]. Moreover, we have established a method to numerically analyze the underlying coupling coefficients between the plasmon mode and molecular vibration modes that allows us to extract the IR absorbance and determine the concentration of molecules (i.e., quantitative measurement). Our device has addressed some critical issues in IR spectroscopy such as quantitative measurement and measurement in an aqueous solution.

Aside from the sensitivity concern, the fabrication of IR-compatible devices is a major obstacle to applications of IR absorption spectroscopy in micro/nanofluidics. In conventional micro/nanofluidic devices, glass or dimethylpolysiloxane (PDMS) are commonly used as substrate materials, while for IR measurement, IR-transparent materials such as calcium fluoride, sapphire, zinc selenide, or silicon are necessary, since the device should be able to transmit IR light, or at least possess an IR transparent window. At that time, bonding is another critical issue that needs to be addressed. Calcium fluoride (CaF_2_) is an ideal candidate substrate for IR spectroscopy, since it has a wide transmission range (i.e., 25,000 to 800 cm^−1^) and a relatively strong chemical tolerance, compared to other IR-transparent materials. To achieve well-defined nanometer-scale structures of nanofluidic devices, lithography followed by the direct cutting or milling of materials is a standard process, but it is quite difficult to physically etch CaF_2_ by conventional techniques such as inductively coupled plasma etching, reactive-ion etching, fast-atom-beam etching, or even focus ion beam milling. Several CaF_2_ microfluidic device configurations employing molten wax [20], negative epoxy photoresist [21,22], or PDMS [23] thin film as adhesive layers, have been demonstrated for IR imaging and spectroscopy. Here, the microchannels were printed on polymer thin films, which were then sandwiched between two CaF_2_ substrates as adhesive layers. Although this strategy offers microfluidic devices with a prominent IR-transparent window, the controllability of dimensions and geometries of fabricated fluidic channels is limited at a 10–100 μm scale. Several groups have reported the wet-etching of CaF_2_ to generate fluidic channel patterns, but the crystalline structures of CaF_2_ usually induce the anisotropic etching, results in rough surfaces with micrometer-scale grains [24]. The fabrication strategies reported so far are not applicable for nanofluidic devices, in which the geometry of channels should be controlled at nanoscale precision. In previous work, we have reported the direct bonding of CaF_2_ and SiO_2_ substrates at room temperature by surface activated bonding. The CaF_2_ substrate was treated with UV/O_3_ to generate the -OH groups on the surface, that formed covalent bonds with silanol groups (Si-OH) activated on the SiO_2_ substrate. Although the post-bonded device was confirmed to tolerate an applied pressure of up to 200 kPa, the bonding strength is not sufficient to perform a continuous fluidic operation due to the gradual degradation of -OH on CaF_2_.

In this study, we propose a robust and durable bonding of CaF_2_ and SiO_2_ at room temperature and realize a nanofluidic device configuration for IR absorption spectroscopy in the near- and mid-IR regime (8000–800 cm^−1^) by employing a thin SiOx film as a cushion layer between the CaF_2_ and SiO_2_ substrate. The nanofluidic channel is fabricated on a SiO_2_ substrate, and then bonded with a CaF_2_ substrate covered with a 10 nm-thick SiOx layer. The nanochannel on SiO_2_ is decorated with an Au mirror as a reflector that allows the measurement in the transflection mode, in which the IR light is transmitted through the upper CaF_2_ substrate and samples, reflects off the mirror, then passes through the samples and substrate again. Moreover, we demonstrate the integration of MIM structures into the nanochannels and perform a proof-of-concept of plasmon-enhanced infrared absorption spectroscopy in the mid-IR regime below 2000 cm^−2^, which cannot be achieved by previously reported devices using SiO_2_ substrates.

## 2. Theory of Metal-Insulator-Metal (MIM) Perfect Absorber Metamaterial Structures for Plasmon-Enhanced Infrared Absorption Spectroscopy

**Figure 1:**
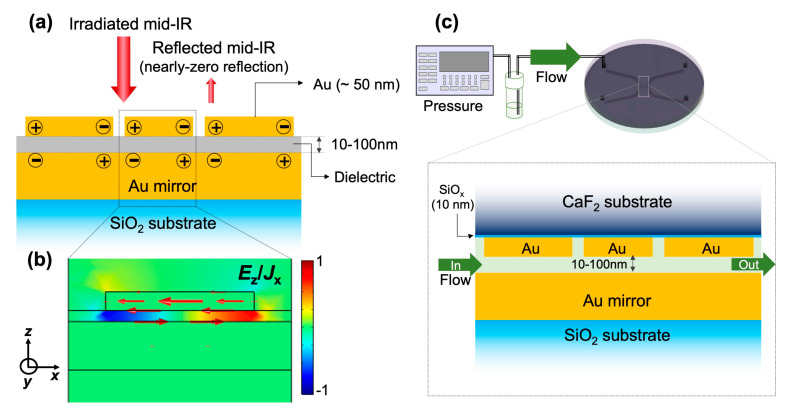
(**a**) Conceptual diagram of metal-insulator-metal (MIM) perfect absorber metamaterial structures for plasmon-enhanced infrared absorption spectroscopy, (**b**) profiles of electric field *E*_z_ and current density *J*_x_ indicated by red arrows elucidating the formation of the quadrupole resonance mode, and the trapping of near-field energy within the nanogap, and (**c**) conceptual diagram of nanofluidic device integrated with MIM perfect absorber structures.

In general, the MIM metamaterial structure consists of an array of Au nanostructures on a thick Au film (i.e., Au mirror) separated by a dielectric gap layer, as shown in Figure 1a [17,18,19,25,26]. Under a normal incident irradiation of IR light, there is a strong plasmonic resonant mode originating from the antiparallel currents excited in the Au nanostructures and the Au mirror layer, forming the quadrupole resonance (i.e., the magnetic dipole resonance mode). The numerical calculation profiles of electric field *E*_z_ and current density *J*_x_ of the corresponding system carried out by the finite-element method (FEM) in Figure 1b obviously reveal the electric dipole on the Au nanostructures and the induced opposite dipole on the mirror. It should be noticed that at resonance, the enhanced electric field is localized in the nanogaps defined by the Au nanostructures and Au mirror. In other words, the near-field energy is efficiently trapped between two layers of Au. This quadrupole mode is a non-radiative one, therefore, no light is reflected back. Since the mirror layer blocks all the transmitted light, the combination of nearly-zero reflectance (R) and transmittance (T) results in the perfect absorption of light. These structures, which are also known as a perfect absorber metamaterial, that not only offer a large plasmonic enhancement, but also allow a low-background detection scheme to gain a high detection performance [25].

The integration of MIM structures into nanofluidic devices by replacing the dielectric gap with a nanofluidic channel is depicted in Figure 1c. The nanochannel is embedded with an array of Au nanostructures on the top wall and a thick Au mirror on the bottom one, while the nanogap between two layers of Au is controlled at several tens of nanometers. This configuration enables the delivery of target molecules into the hot-spots of MIM perfect absorber structures by fluidic operation, thus significantly improving the performance of SEIRA. Since CaF_2_ is used in the upper substrate, it is possible to measure throughout the near- and mid-IR regime (8000–800 cm^−1^) in the transflection mode. Most of the MIM perfect absorber structures and their applications reported so far have focused on a single wavelength where the plasmon resonance is excited in one type of nanostructures. In this study, we also demonstrate a proof-of-concept of a multi-band perfect absorber towards multispectral plasmon-enhanced infrared absorption spectroscopy. It is based on a combination of several sizes of nanostructures in the unit cell. Several bands of large absorption are experimentally accomplished, and those bands can be readily tuned throughout the mid-IR regime.

## 3. Materials and Methods

### 3.1. Materials

Several ϕ50.0 and 0.7 mm-thick SiO_2_ (BK7) and IR-grade CaF_2_ substrates were purchased from OptoSigma (Tokyo, Japan). All substrates were double-side polished to the optical grade. Drilled on the CaF_2_ substrate were ϕ0.7 mm through-holes at specific positions in advance as inlets and outlets for introducing fluidics. Those holes were also used as markers for position alignment. The SiO_2_ substrate was cleaned in a piranha solution (H_2_SO_4_/H_2_O_2_ = 3:1) before fabrication. In the case of CaF_2_ substrate, the UV/O_3_ treatment was performed instead of piranha washing. The deposition of SiO_2_ and MgF_2_ thin films described in Section 3.2.2 and Section 4.3, respectively used source materials that were purchased from Furuuchi Chemical Corp. Sulfuric acid, hydrogen perroxide, acetone, and deuterium oxide of special grade were purchased from Nacalai Tesque (Kyoto, Japan).

### 3.2. Methods

#### 3.2.1. Fabrication of Fluidic Channels on the Bottom of the SiO_2_ Substrate

**Figure 2:**
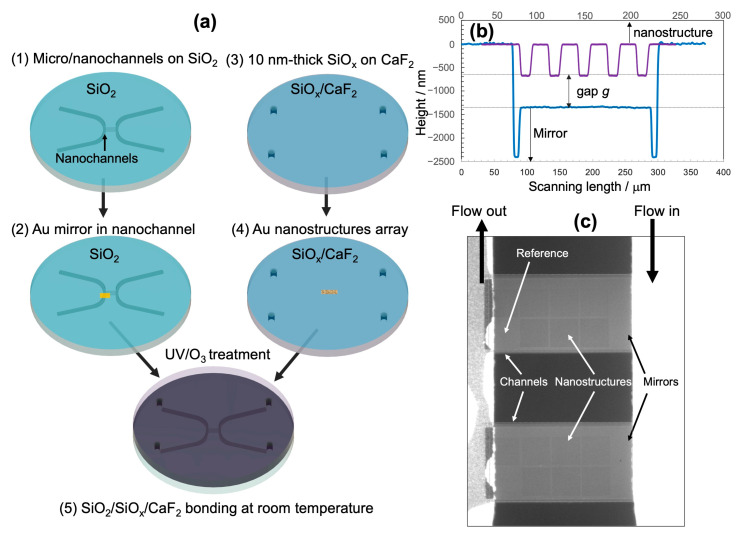
(**a**) Fabrication process of the infrared (IR)-compatible nanofluidic device integrated with MIM perfect absorber metamaterials, (**b**) nanogap *g* calculated from the height profiles of the bottom nanochannel and top nanostructure, and (**c**) fluorescence image of nanochannels confirming the success of bonding.

The fabrication process is described in Figure 2a. Large microchannels (designed width: 400 μm; depth: 5 μm) were patterned and written on a SiO_2_ substrate by UV photolithography using maskless photolithography equipment (NanoSystem Solutions Inc., DL-1000, Okinawa, Japan) and AZ-1500 photoresist (MicroChemicals, Ulm, Germany), followed by reactive ion etching in CF_4_/O_2_ gas (SAMCO, RIE-200P, Kyoto, Japan). Nanochannel patterns (designed width: 220 μm; length: 400 μm) were patterned on the same substrate by UV photolithography. A careful alignment was necessary to ensure that nanochannels were bridging the inlet and outlet microchannels. After developing, the substrate was immersed into a solution of 1.25% buffered hydrofluoric acid (Sigma-Aldrich Japan, Tokyo, Japan). The depth of the nanochannels was finely controlled by the immersion time, with an approximate etching rate of 0.7 nm/s. The nanochannel depth, which was usually on 200–250 nm, was determined according to the desired value of the final nanogap *g*. After that, the substrate was cleaned again in a piranha solution to remove the contaminants. Finally, the Au mirror layer was fabricated inside the nanochannel by UV photolithography, followed by the deposition of a 3 nm-thick Cr (adhesive layer), and an Au thin film thicker than 100 nm. After lift-off, it generated a mirror layer inside the nanochannel. It should be noted that the designed width of the mirror pattern is 200 μm, which is smaller than that of the nanochannel, and the accuracy in position alignment using the maskless UV lithography equipment allows the fabrication of mirror layers inside the nanochannels.

#### 3.2.2. Fabrication of Nanostructures on Top of the CaF_2_ Substrate

After the treatment in UV/O_3_, the CaF_2_ substrate was deposited with a 10 nm-thick layer of SiO*_x_* by employing a custom-built electron beam evaporator by KATAGIRI Engineering (Kanagawa, Japan) using a commercial SiO_2_ source. The thickness was controlled by a quartz crystal monitor. The IR absorption spectrum of the post-deposited substrate verified the existence of the SiO_x_ layer. The substrate was then coated with a 150 nm-thick ZEP-520A resist (Zeon Corp., Tokyo, Japan). To prevent the exfoliation of the SiO*_x_* layer due to the difference in thermal expansion coefficients of SiO_x_ and CaF_2_, the pre-bake of resist was performed in an electric furnace with a very slow temperature rise (3 °C/min) and natural cooling. After pre-baking at 120 °C in 30 min, the resist layer was coated with a thin layer of conducting polymer Espacer 300Z (Showa Denko, Tokyo, Japan) to avoid the charge accumulation during the electron beam lithography (EBL). Nano square-disk patterns were written by EBL (Elionix, ELS-7700, Tokyo, Japan). After developing in a methyl butyl ketone solvent, the substrate was then deposited with a 3 nm-thick Cr (adhesive layer) and 60 nm-thick Au film by a custom-built thermal evaporator by KATAGIRI Engineering. After lift-off in anisole solvent under the assistance of sonication, the achieved Au nano square-disk patterns were observed and analyzed by scanning electron microscope (Hitachi High-Tech, SU8010, Tokyo, Japan) under an acceleration voltage of 5.0 kV. The size of each nanostructure array was 80 × 80 μm^2^.

#### 3.2.3. Bonding of the Device

In the surface activated bonding of SiO_2_ substrates, the heating at 100–200 °C is usually applied to generate the dehydration reaction of silanol groups, forming covalent bonds of siloxane. In our case, when the heating was applied, the bonding was failed. It may be caused by the exfoliation of the deposited SiO_x_ layer from the CaF_2_ substrate due to the large difference in the thermal expansion coefficients of SiO_2_ and CaF_2_. The temperature control is eventually a key factor in the SiO_2_/CaF_2_ bonding. Herein, the two substrates underwent the UV/O_3_ treatment in 30 min with a controlled exposure and cooling time so that the temperature of the substrates did not exceed 35 °C. Right before the bonding, the substrates were cleaned in an ultrapure water to fully activate the −OH groups on the surfaces. After completely drying under air flow, the alignment of the top square-disk and bottom mirror was carried out under a 4× microscope. The nanogap *g* was calculated based on the depth of the original nanochannel, the height of the mirror layer, and the height of nanostructures, which were measured by a surface profiler (KLA-Tencor, P16, Milpitas, CA, USA) (Figure 2b). Since the nanostructure is too small to probe by surface profiling, we fabricated large grating patterns on the same substrate, and used them to determine the height of the nanostructures. They were also used as markers for alignment in bonding.

### 3.3. IR Reflectance Measurement

The reflectance property was characterized by using a Fourier-transformed infrared spectrometer (JASCO, FT/IR-6300, tokyo, Japan) equipped with a microscope (JASCO, IRT-5200). All spectra were measured at a normal incident angle. A liquid nitrogen-cooled high-sensitive MCT (HgCdTe, Thorlabs, Newton, NJ, USA) detector was used with a frequency resolution of 4 cm^–1^.

### 3.4. Numerical Calculation

To clarify the underlying plasmonic resonant modes and the field distribution of the corresponding system, a set of numerical simulations was carried out by the finite-element method using a commercial software package (Multiphysics, COMSOL, Burlington, MA, USA). The Cr adhesive layer presented in the experimental samples was omitted in the simulations.

### 3.5. Fluidic Operation

The injection of the sample solution was carried out by the pressure-driven flow. A stable flow of dried nitrogen gas was introduced into a vial containing the target solution by employing a pressure controller (Nagano Keiki, PC20, Tokyo, Japan). All solutions were introduced into the nanochannels by a pressure-driven flow of 300 kPa, and the device was then measured at a static state (no-flow condition). When a new solution was introduced, the flow was kept for more than 30 min to ensure that the molecules inside the channel were completely substituted.

## 4. Results

### 4.1. Leakage Test

A fluorescein solution (2 × 10^−4^ M) was introduced at an applied pressure up to 300 kPa to investigate the bonding condition. The fluorescence image of the nanochannels in Figure 2c confirmed no leakage of the liquid. It ensures that the bonded microchip could be used as a nanofluidic device using a pressure-driven flow. Since the inner interfaces of nanochannels are SiO_2_ and Au, our nanofluidic device showed a good chemical tolerance to common organic solvents, and a neutral or acidic aqueous solution. However, we observed the leakage of the device when a rapid change in temperature was applied, due to the exfoliation of a 10 nm-thick SiO_x_ adhesive layer. Temperature management is thus critical for handling this kind of IR-compatible nanofluidic device.

### 4.2. IR-Compatible Nanofluidic Device Integrated with MIM Metamaterials and Proof-of-Concept of Detection of Molecules

**Figure 3:**
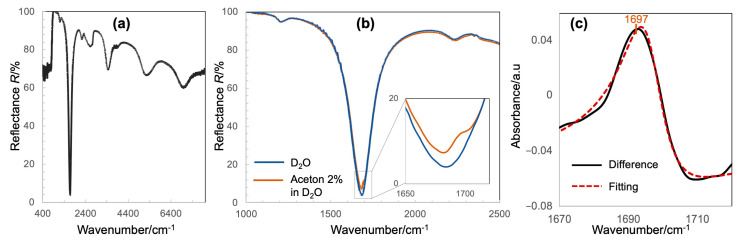
(**a**) Typical reflectance spectrum of the IR-compatible nanofluidic device integrated with MIM perfect absorber metamaterial, (**b**) reflectance spectra when the device is filled with D_2_O and acetone/D_2_O, respectively revealing the IR absorption signal of C═O, and (**c**) the Fano line-shape fitting of the difference spectrum for retrieving the intrinsic IR absorption of C═O. (**a**) Typical reflectance spectrum of the IR-compatible nanofluidic device integrated with MIM perfect absorber metamaterial, (**b**) reflectance spectra when the device is filled with D_2_O and acetone/D_2_O, respectively revealing the IR absorption signal of C═O, and (**c**) the Fano line-shape fitting of the difference spectrum for retrieving the intrinsic IR absorption of C═O.

Figure 3a shows a typical reflectance spectrum of the fabricated device taken at MIM structures when the nanochannel is filled with D_2_O. The spectrum is measured in the transflection mode, and is referenced to the channel at the Au mirror, as described in the fluorescence image in Figure 2c. The spectrum exhibits several reflectance dips, and the strongest among them can be attributed to the above-mentioned quadrupole resonance, at which the reflected light is almost eliminated. Since the bottom Au film is thick enough to block the transmitted light, the zero transmission and cancellation of reflection implies the perfect absorption. Although the upper substrate is covered with a 10 nm-thick SiO_x_ layer, the device exhibits a prominent transparency throughout the IR regime ensuring its performance as an IR-compatible nanofluidic device. The result also verifies the possibility of finely tuning the plasmon resonance to the molecular vibrational modes of interests.

Using this device, we demonstrated the proof-of-concept detection of acetone molecules. The device was purposely designed so that its quadrupole plasmon resonance spectrally overlapped with the C═O stretching mode of acetone at ~1700 cm^−^^1^, when the device is filled with D_2_O. D_2_O was used rather than H_2_O to avoid the overlapping with the O–H stretching band. The reflectance spectra when the devices were filled with D_2_O and an acetone 2% solution, respectively in Figure 3a, obviously shows the absorption feature of C═O, which appears as a Fano-like anti-resonance peak within a broad reflectance dip of the original plasmon resonance. There is a slight modification in the absorption line shape and spectral position of C═O vibrational stretch, which is an evidence of a strong interaction between the plasmonic resonances and molecular vibrations. The nanogap of the device used in this experiment is 70 nm, which corresponds to a detection volume of ~50 fL or ~21 fmole of C═O. It should be noted that a strong signal with prominent S/N has been achieved for that ultrasmall amount of molecules. The difference spectrum retrieved from the spectra in Figure 3b also represents a Fano-liked spectral shape, in which the peaks are also slightly off from the general values of the C═O stretching band. To quantitatively analyze the vibrational signal, the Fano line-shape fitting was carried out with the following function form.

(1) $$I\propto \frac{\left(\omega -\omega_{vib}+F\gamma \right)}{{\left(\omega -\omega_{vib}\right)}^{2}+\gamma^{2})}$$

where ω_vib_ is the resonant frequency, *γ* is the damping constant (HWHM), and *F* is the Fano parameter to describe the degree of asymmetry. The spectral line-shapes of the experimental result were well re-produced, and the fitting revealed the underlying vibrational resonance of C═O at 1697 cm^−^^1^ [27]. This value is in good agreement with that of acetone in an aqueous solution [28], indicating the possibility of this device in both the identification and quantitative measurement of molecules. This fitting suggests a new reliable analysis method of molecular-plasmon coupled spectrum, which is simpler than the numerical fitting model based on the temporal coupled theory model (TCTM) reported previously [13,17,18,19].

### 4.3. A Proof-of-Concept of Multi-Band Metal-Insulator-Metal (MIM) Structures towards Multispectral Plasmon-Enhanced Infrared Absorption Spectroscopy

**Figure 4:**
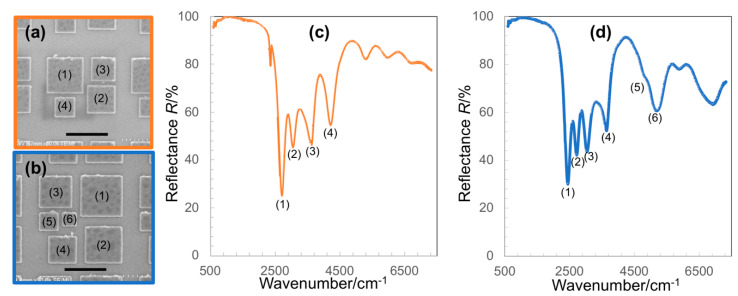
(**a**,**b**) Typical top-view SEM images of multi-band metal-insulator-metal (MIM) structures, (**c**,**d**) reflectance spectra of the corresponding MIM structures in (**a**,**b**) revealing absorbers of multi-bands that can be readily tuned throughout the mid-IR regime. The scale bar in (**a**,**b**) is 1 μm.

As observed in the spectra in Figure 3, although the quadrupole resonance is relatively broader than the sharp vibrational modes of molecules, it is quite difficult to broaden its band to cover several molecular vibrational modes simultaneously. Here, we introduced a multi-band absorber based on the combination of several sizes of nanostructures in a unit cell, and demonstrated a proof-of-concept of multi-band metal-insulator-metal (MIM) structures. Figure 4a,b shows the top-view SEM images of multi-band MIM structures, which are composed of several nano square-disks of different sizes, which are periodically arranged on a substrate consisting of a thick Au film and a 70 nm-thick MgF_2_ layer. The variation in the sizes of nanostructures results in the reflectance spectra with several separate plasmon resonant modes, yielding less than a 50% reflectance at each mode (Figure 4c,d). Each mode corresponds to one size of the nanostructures indicated by the numbers in the SEM images. The larger the nanostructure is, the lower the wavenumber its resonance exhibits. By altering the dimensions of associated geometric parameters, resonance wavelengths can be tuned individually to spectrally overlap with the target vibrational modes of molecules throughout the mid-IR regime. Therefore, these structures are very promising for tracking several characteristic molecular vibrational modes simultaneously, making it possible to identify different molecular species or complex biological entities.

## 5. Discussion

In general, the effective path length in our device is the double-length of nanogap *g*, since the IR light transmits the samples, reflects off the mirrors, and passes through the samples again. However, in a plasmon-molecular coupled system, the effective path length cannot be defined by this definition, since the enhanced electromagnetic field apparently lengthens the temporal interaction of light and molecules. This is the key in amplifying the signals of molecules in case where the spatial interaction of molecules with light is extremely limited. Furthermore, the controllable introduction of target molecules into the most enhanced electromagnetic field of the MIM metamaterial absorber has contributed to the high performance in IR absorption detection. However, it is also important to point out the low-background detection scheme. Here, the molecular vibrational modes appeared as reflected peaks in the low-reflectance background of the plasmon resonance. It contrasts with the conventional reflection absorption infrared spectroscopy (RAIRS), in which the molecular signal is detected as a small dip in a bright and noisy background of metal mirror. This feature becomes significant in molecular sensing using bright IR sources such as synchrotron radiation or QCL lasers. This study has also demonstrated a versatile strategy to realize multi-band perfect absorbers for multispectral IR spectroscopy. An ideal context of the multi-band absorber is the high degree of symmetry in the optical characteristics of the resonant modes to ensure the associated field enhancement effects in all modes at the same level [26]. Although this feature was not obtained in our results, the fine tuning of sizes and shapes of nanostructures are under going to realize this kind of highly symmetric multi-band MIM perfect absorber.

## 6. Conclusions

In this study, we have elucidated the issues involving the fabrication of IR-compatible nanofluidic devices and demonstrated a versatile method to overcome them. It is enabled by the bonding of CaF_2_ and SiO_2_ at room temperature. The bonding strength is confirmed to be strong enough for pressure-driven fluid control, and the fluidic performance in our device is comparable to the ones in conventional nanofluidic devices using SiO_2_ substrates. Our nanofluidic devices exhibit good transparency in a wide range of near- and mid-IR (8000 – 800 cm^−1^). The integration of MIM metamaterials into nanofluidic devices has allowed the controllable delivery of molecules into the hot-spots of metamaterials, thus enhancing the resonant coupling between molecules and plasmonic structures. It results in a significant improvement in sensing of molecular IR absorption, compared to conventional MIM structures. Moreover, although further investigation is required to optimize the structures, this study has introduced an easy fabrication approach for the multi-band perfect absorber. These kinds of structures could find numerous potential applications aside from multispectral plasmon-enhanced infrared absorption spectroscopy, such as selective thermal emitters, micro-bolometers, as well as photovoltaic and thermo-photovoltaic cells.

## References

[1] Elvira K.S., Solvas X.C.i., Wootton R.C.R., de Mello A.J. (2013). The past, present and potential for microfluidic reactor technology in chemical synthesis. Nat. Chem..

[2] Tsukahara T., Mawatari K., Kitamori T. (2010). Integrated extended-nano chemical systems on a chip. Chem. Soc. Rev..

[3] Monat C., Domachuk P., Eggleton B.J. (2007). Integrated optofluidics: A new river of light. Nat. Photon..

[4] Aroca R. (2006). Surface Enhanced Vibrational Spectroscopy.

[5] Movasaghi Z., Rehman S., Ihtesham R. (2008). Fourier transform Infrared (FTIR) spectroscopy of biological tissues. Appl. Spectrosc. Rev..

[6] Barth A. (2007). Infrared spectroscopy of proteins. Biochim. Biophys. Acta. Bioenergy.

[7] Diem M., Romeo M., Boydston-White S., Miljkovic M., Matthaus C. (2004). A decade of vibrational micro-spectroscopy of human cells and tissue. Analyst.

[8] Loutherback K., Birarda G., Chen L., Holman H.-Y.N. (2016). Microfluidic approaches to synchrotron radiation-based Fourier transform infrared (SR-FTIR) spectral microscopy of living biosystems. Protein Pept. Lett..

[9] Vaccari L., Birarda G., Businaro L., Pacor S., Grenci G. (2012). Infrared microspectroscopy of live cells in microfluidic devices (MD-IRMS): Toward a powerful label-free cell-based assay. Anal. Chem..

[10] Andrew Chan K.L., Gulati S., Edel J.B., de Mello A.J., Kazarian S.G. (2009). Chemical imaging of microfluidic flows using ATR-FTIR spectroscopy. Lab Chip.

[11] Adato R., Yanik A.A., Amsden J.J., Kaplan D.L., Omenetto F.J., Hong M.K., Erramilli S., Altug H. (2009). Ultra-sensitive vibrational spectroscopy of protein monolayers with plasmonic nanoantenna arrays. Proc. Natl. Acad. Sci. USA.

[12] Bomers M., Charlot B., Barho F., Chanuel A., Mezy A., Cerutti L., Gonzalez-Posada F., Taliercio T. (2020). Microfluidic surface-enhanced infrared spectroscopy with semiconductor plasmonics for the fingerprint region. React. Chem. Eng..

[13] Adato R., Altug H. (2013). In-situ ultra-sensitive infrared absorption spectroscopy of biomolecule interactions in real time with plasmonic nanoantennas. Nat. Commun..

[14] Rodrigo D., Tittl A., Ait-Bouziad N., John-Herpin A., Limaj O., Kelly C., Yoo D., Wittenberg N.J., Oh S., Lashuel H.A. (2018). Resolving molecule-specific information in dynamic lipid membrane processes with multi-resonant infrared metasurfaces. Nat. Commun..

[15] Le H.H.T., Morikawa K., Shimizu H. (2020). Advances in label-free detections for nanofluidic analytical devices. Micromachines.

[16] Fan X., White I.M. (2011). Optofluidic microsystems for chemical and biological analysis. Nat. Photon..

[17] Le H.H.T., Tanaka T. (2017). Plasmonics-nanofluidics hydrid metamaterial: An ultrasensitive platform for infrared absorption spectroscopy and quantitative measurement of molecules. ACS Nano.

[18] Le H.H.T., Morita A., Mawatari K., Kitamori T., Tanaka T. (2018). Metamaterials-Enhanced Infrared Spectroscopic Study of Nanoconfined Molecules by Plasmonics-Nanofluidics Hydrid Device. ACS Photonics.

[19] Le H.H.T., Morita A., Tanaka T. (2020). Refractive index of nanoconfined water reveals its anomalous physical properties. Nanoscale Horiz..

[20] Chan K.L.A., Niu X., de Mello A.J., Kazarian S.G. (2010). Rapid prototyping of microfluidic devices for integrating with FT-IR spectroscopic imaging. Lab Chip.

[21] Kulka S., Kaun N., Baena J.R., Frank J., Svasek P., Moss D., Vellekoop M.J., Lendl B. (2004). Mid-IR synchrotron radiation for molecular specific detection in microchip-based analysis systems. Anal. Bioanal. Chem..

[22] Polshin E., Verbruggen B., Witters D., Sels B., DeVos D., Nicolaï B., Lammertyn J. (2014). Integration of microfluidics and FT-IR microscopy for label-free study of enzyme kinetics. Sens. Actuators B.

[23] Perro A., Lebourdon G., Henry S., Lecomte S., Servanta L., Marre S. (2016). Combining microfluidics and FT-IR spectroscopy: Towards spatially resolved information on chemical processes. React. Chem. Eng..

[24] Lehmkuhl B., Noblitt S.D., Krummel A.T., Henry C.S. (2015). Fabrication of IR-transparent microfluidic devices by anisotropic etching of channels in CaF_2_. Lab Chip.

[25] Ishikawa A., Tanaka T. (2015). Metamaterial absorbers for infrared detection of molecular self-assembled monolayers. Sci. Rep..

[26] Chen K., Adato R., Altug H. (2012). Dual-band perfect absorber for multispectral plasmon-enhanced infrared spectroscopy. ACS Nano.

[27] Cerjan B., Yang X., Nordlander P., Halas N.J. (2016). Asymmetric aluminum antennas for self-calibrating surface-enhanced infrared absorption spectroscopy. ACS Photonics.

[28] Max J.-J., Chapados C. (2003). Infrared spectroscopy of acetone–water liquid mixtures. I. Factor analysis. J. Chem. Phys.

